# Cytokine Signature of Dengue Patients at Different Severity of the Disease

**DOI:** 10.3390/ijms22062879

**Published:** 2021-03-12

**Authors:** Irwin Puc, Tzu-Chuan Ho, Ko-Lun Yen, Amrita Vats, Jih-Jin Tsai, Po-Lin Chen, Yu-Wen Chien, Yu-Chih Lo, Guey Chuen Perng

**Affiliations:** 1 Institute of Basic Medical Sciences, College of Medicine, National Cheng Kung University, Tainan 701401, Taiwan; irwin.i.puc@gmail.com (I.P.); anywayhowtodo@gmail.com (T.-C.H.); abcabc623@gmail.com (K.-L.Y.); amritaicmr@gmail.com (A.V.); 2 Tropical Medicine Center, Kaohsiung Medical University Hospital, Kaohsiung 807378, Taiwan; jijits@cc.kmu.edu.tw; 3 Division of Infectious Diseases, Department of Internal Medicine, Kaohsiung Medical University Hospital, Kaohsiung 807378, Taiwan; 4 School of Medicine, College of Medicine, Kaohsiung Medical University, Kaohsiung 807378, Taiwan; 5 Department of Internal Medicine, College of Medicine, National Cheng Kung University, Tainan 701401, Taiwan; cplinmayer@gmail.com; 6 Department of Public Health, College of Medicine, National Cheng Kung University, Tainan 701401, Taiwan; yuwenchien@mail.ncku.edu.tw; 7 Department of Occupational and Environmental Medicine, National Cheng Kung University Hospital, College of Medicine, National Cheng Kung University, Tainan 701401, Taiwan; 8 Department of Biotechnology and Bioindustry Sciences, College of Bioscience and Biotechnology, National Cheng Kung University, Tainan 701401, Taiwan; 9 Department of Microbiology and Immunology, College of Medicine, National Cheng Kung University, Tainan 701401, Taiwan

**Keywords:** dengue, cytokines, biomarker prediction, severe dengue, flaviviruses

## Abstract

Clinical presentations of dengue fever (DF) are diverse and non-specific, causing unpredictable progression and outcomes. Its progression and severity have been associated with cytokine levels alteration. In this study, dengue patients were classified into groups following the 2009 WHO dengue classification scheme to investigate the cytokine signature at different severity of the disease: dengue without warning sign symptoms (A); dengue with warning signs (B); severe dengue (C); other fever (OF) and healthy (Healthy). We analyzed 23 different cytokines simultaneously, namely IL-1b, IL-2, IL-4, IL-6, IL-8, IL-10, IL-12p70, IL-17A, IL-33, CD14, CD54, CD62E, CD62L, CD62p, CD106, CD121b, CD154, CD178, GM-CSF, IFN-g, MIF, ST2 and TNF from patients admitted to National Cheng Kung University Hospital during the 2015 Taiwan dengue outbreak. Cytokines TNF, CD54, CD62E, CD62L, CD62P, GM-CSF, IL-1b, IL-2, IL-6, IL-8, IL-10, IL-12p70, IL-17A, INF-g and MIF were elevated while CD106, CD154, IL-4 and L-33 were decreased when compared to the control. IL-10 demonstrated to be a potential diagnostic marker for DF (H and A group; AUC = 0.944, H and OF group; AUC = 0.969). CD121b demonstrated to be predictive of the SD (A and B group; AUC = 0.744, B and C group; AUC = 0.775). Our results demonstrate the cytokine profile changes during the progression of dengue and highlight possible biomarkers for optimizing effective intervention strategies.

## 1. Introduction

Dengue is currently regarded as the most common arthropod-borne viral disease transmitted by *Aedes* mosquito in tropical and subtropical areas. Its incidence has increased more than 30 folds in recent years along with the geographic expansion of its *Aedes* vector mosquito [1]. Globally, it is estimated that 3.9 billion people from more than 128 countries are at risk of dengue virus (DENV) infection, with 284–528 million cases occurring each year, of which 96 million manifest clinically with a severe form of the disease [2,3]. World Health Organization (WHO) reports that approximately 500,000 people with severe dengue require hospitalization each year, and about 2.5% of those affected die [4]. Despite dengue being first isolated more than 70 years ago and affecting almost half of the world’s population, to date, there is no protective vaccine or effective treatments available [5]. The lack of such a “cure” can be attributed to our incomplete understanding of dengue immunopathogenesis, lack of a suitable animal model that would mimic similar clinical symptoms as patients and the inherent dangers of seronegative subjects receiving live-attenuated dengue vaccines [6].

DENV is a positive-sense single-stranded RNA whose genome is 11 kb in length which encodes three structural (capsid, membrane, envelope) and seven non-structural (NS1, NS2A, NS2B, NS3, NS4A, NS4B and NS5) proteins [7]. There are four distinct serotypes of DENV. Dengue exhibits symptoms ranging from mild fever or flu-like illness, typical dengue fever (DF) to potentially lethal severe dengue (SD), dengue hemorrhage fever (DHF) or dengue shock syndrome (DSS). However, there are no specific features used to distinguish between acute mild dengue and severe dengue because each serotype of DENV can cause nearly identical clinical manifestations in humans as well as circulate in the same niche [8,9]. Moreover, acute dengue has shown symptoms ranging from high fever, myalgia and headaches which are also observed in Zika and Chikungunya virus infections [10,11]. The mechanism by which only a small portion of dengue-infected patients go on to develop a more severe form of the disease remains a baffling mystery and has been a subject of intense study and debate over recent years [3,12,13]. The risk to develop SD is plausibly due to many factors, dengue serotype, secondary infection by a heterologous serotype, age, comorbidity, poor clinical prognosis, diagnosis, virulence and the host immune response [14,15].

Even though several risk factors for the progression of SD have been proposed, our complete understanding of the pathogenesis of SD remains unknown. Regardless, one of the well-known hypotheses that might explain the reason why a secondary DENV infection leads to SD complication is antibody-dependent enhancement [16,17]. Another hypothesis on the progression of SD is the phenomenon known as a cytokine storm, which is the alteration of cytokine and chemokine levels that cause the endothelial cells to malfunction and eventually lead to vascular permeability of endothelial cells and plasma leakage as seen in DHF and DSS [18]. Cytokines and chemokines are small proteins ranging from 8 to 40 kDa, and they are cell signaling molecules that interact with each other to orchestrate a variety of functions including cell growth, proliferation, differentiation, maturation and immunity [10,11]. Numerous studies have demonstrated that the concentrations of cytokines, mediators and soluble receptors are significantly increased during DENV infection, this dysregulation of certain cytokines has major implications in dengue pathogenesis especially in DHF and DSS [19,20,21,22]. By now, it is common knowledge that DENV can infect various immune cells like dendritic cells and monocytes which contribute to the production of inflammatory cytokines. This increased unregulated production of cytokines can aggravate pathogenesis, organ failure and cause death [23].

As such, dengue possesses a significant challenge in clinical management and detection especially during an outbreak, mainly due to poor prognostics in being able to differentiate between individuals who have DF from those who might progress to SD or sometimes even may result in over-hospitalization depending on the criterion used for hospital admission [24]. For this reason, the cytokine storm has been studied to try and distinguish potential patterns or biomarkers that can be used to detect SD. Using these specific cytokine profile patterns would allow us to have a better understanding of the progression of dengue pathogenesis, allowing quicker diagnosis to predict patients who would likely proceed to SD and reducing the mortality rate.

Therefore, in this study, we aimed to explore the relationship between cytokines levels at different phases of dengue fever with an objective to identify a biomarker that could help in distinguishing dengue fever from SD or the progression of the disease.

## 2. Results

### 2.1. Characteristics of Cytokine Distribution

A total of 328 cytokine levels were observed and analyzed from 243 patients’ sera to investigate the changes in cytokine profiles during different phases of DENV infection. This is a retrospective study and the complete demographic descriptions of the enrolled patients had been previously reported. In his cytokine study, available data demographic characteristics were shown in Table 1, the mean age of the population was 59.94 (range 18–93 years, standard deviation 19.55). Patients were classified according to the 2009 WHO dengue classification guidelines into dengue without warning sign symptoms (A) which consisted of 128 patients; dengue with warning signs which consisted of 103 (B) and severe dengue (C) which consisted of 53 patients. Eight patients who had fever but were dengue negative were classified as other fever (OF). The healthy (Healthy) control group consisted of 36 patients. There were significant overlaps within the cytokine levels among the group. Some of the cytokines in fever people were found to be lower than healthy controls, while others were found to be significantly higher in dengue patients compared to the healthy controls even though variations among different categories of dengue and OF were observed.

### 2.2. Cytokine Profile in Dengue Patients and Healthy Group

**Figure 1:**
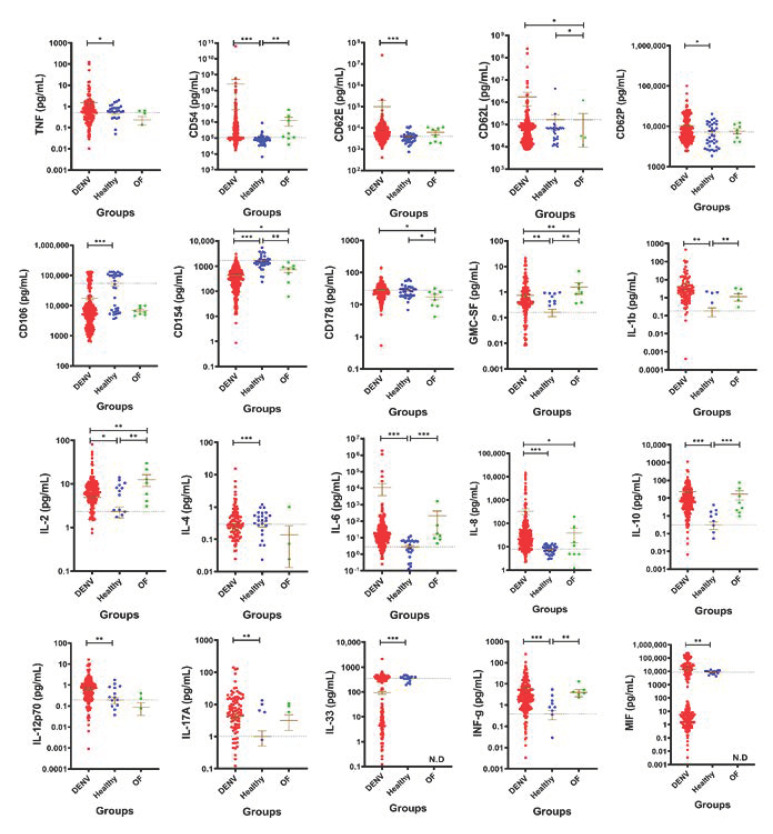
The levels of cytokines in DENV patients. Patients were divided into three groups: Laboratory confirmed DENV-2 patients (DENV), laboratory confirmed DENV negative (OF) and healthy volunteers (Healthy). Only cytokines having a significance difference between either DENV, Healthy or OF were shown here. Cytokines with no significance difference can be found in Supplementary Figure S1. Mean ± SEM. Horizontal dotted line represents health mean average. N.D (no data). *p* < 0.05 *; *p* < 0.01 **; *p* < 0.001 ***.

Our first priority was to compare the levels of IL-1b, IL-2, IL-4, IL-6, IL-8, IL-10, IL-12p70, IL-17A, IL-33, CD14, CD54, CD62E, CD62L, CD62p, CD106, CD121b, CD154, CD178, GM-CSF, IFN-g, MIF, ST2 and TNF in plasma samples of dengue group, OF group and Healthy group. There was a clear indication that the immune system was activated since there was a noticeable change in the expression of most of the Pro and Anti-inflammatory cytokines when compared to the healthy control Figure 1. Cytokine profile analysis revealed a statistically significant difference in the levels of TNF (*p* = 0.0046), CD54 (*p* < 0.001), CD62E (*p* < 0.001), CD62P (*p* = 0.01), GM-CSF (*p* = 0.0080), IL-1b (*p* = 0.0065), IL-2 (*p* = 0.0159), IL-6 (*p* < 0.001), IL-8 (*p* < 0.001), IL-10 (*p* < 0.001), IL-12p70 (*p* < 0.001), IL-17A (*p* = 0.0310), INF-g (*p* < 0.001) and MIF (*p* = 0.0005) when compared to the healthy control. A significant decrease in the levels of CD106 (*p* < 0.001), CD154 (*p* < 0.001), IL-4 (*p* < 0.001) and L-33 (*p* < 0.001) was observed when compared to the healthy control. It was noted that only cytokines CD154, GM-CSF and IL-2 had a statistically significant difference between the three groups (DENV, Healthy and OF), however, CD62L (*p* = 0.03), CD178 (*p* = 0.0293) and IL-8 (*p* = 0.0498) also differentiated DENV from patients with OF. There was no significant difference between any of the groups for cytokines CD14, CD121b and ST2 Supplementary Figure S1. Our results suggest that these group of cytokines can be used as a potential marker to distinguish between dengue fever and OF.

### 2.3. Cytokine Profile in Dengue Patients of Different Severity

**Figure 2:**
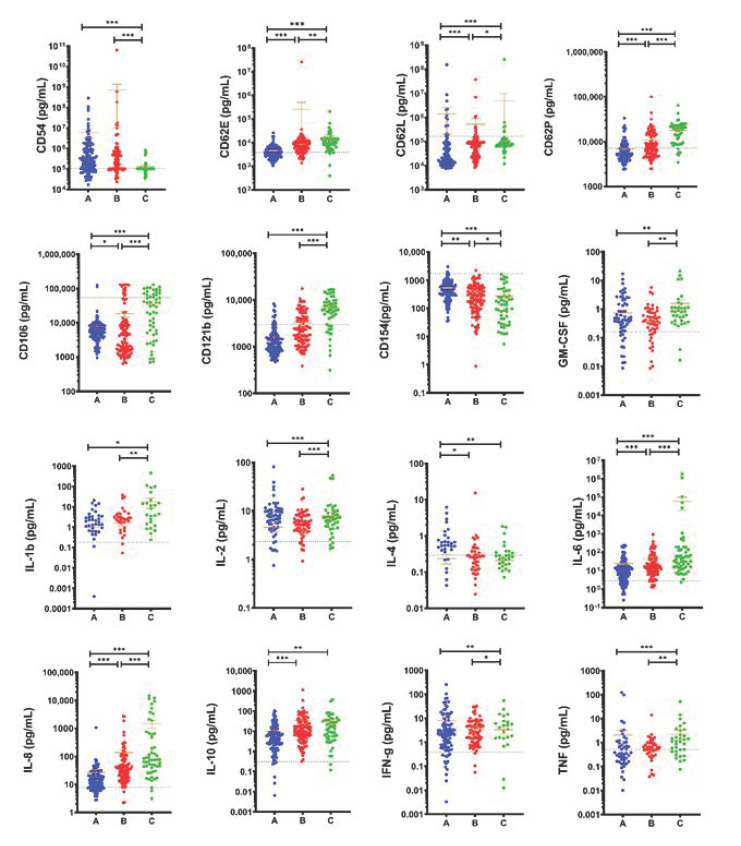
The levels of cytokines in DENV patients with different severity. DENV patients were divided into three groups according to the day of illness and clinical symptoms following the 2009 WHO dengue classification scheme: dengue without warning sign symptoms (A), dengue with warning signs (B) and severe dengue (C). Only cytokines having a significance difference between either A, B or C were shown here. Remaining cytokines can be found in Figure 2. Mean ± SEM. Horizontal dotted line represents health mean average. *p* < 0.05 *; *p* < 0.01 **; *p* < 0.001 ***.

Since the involvement of cytokines is hypothesized to be involved in the progression of SD, we explored the profile change in the levels of cytokines in dengue patients at the different severity of the disease. It was clearly observed that almost all cytokines expression levels were increased when compared to the healthy control mean (baseline) Figure 2. Cytokine profile analysis revealed a significant difference in the levels of CD62E (*p* < 0.001), CD62P (*p* < 0.001), CD106 (*p* = 0.0333), CD121b (*p* < 0.001), IL-6 (*p* < 0.005) and IL-8 (*p* < 0.001). Not only did these cytokines reveal a significant difference between the different groups (A, B and C), but also showed an increased pattern expression along with the disease severity Figure 2. CD154 (*p* < 0.001) also showed a significant difference between the different groups (A, B and C), but exhibited a decreasing pattern along with the disease severity Figure 2. CD62L (*p* < 0.001) showed a significant difference between the different groups but did not show any pattern along with the disease severity. Even though IL-1b and IL-10 showed a visible increase pattern along with the disease severity there was no significant difference between the A and B groups, and the B and C groups, respectively. IL-4 and IFN-g also showed a decreasing pattern along with the disease severity but showed no differences in statistical analysis between B and C and A and B groups, respectively. Of note, there was no significant difference for cytokines CD14, CD176, IL-12p70, IL-17A, IL-33, MIF and ST2 between any of the groups Supplementary Figure S2.

### 2.4. Cytokine Profile of Dengue Patients at Different Days of Fever

**Figure 3:**
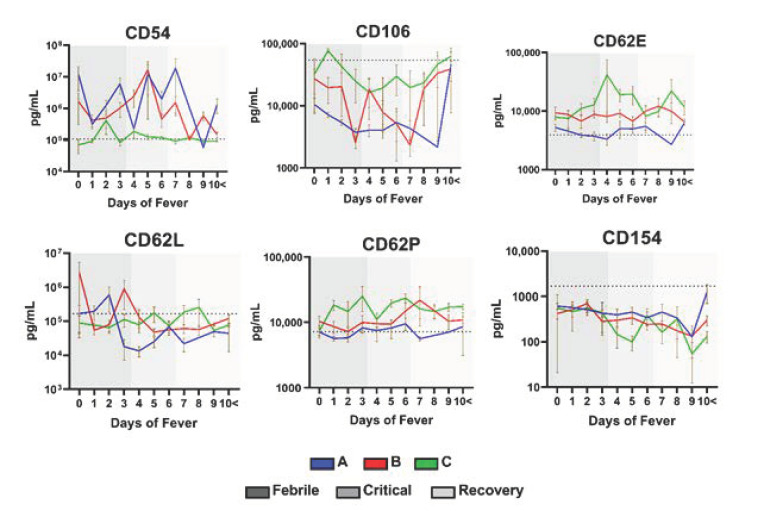
The level of Adhesion molecules in dengue patients at different days of fever. Graphs representing the levels between the different groups of DENV patients previously divided following the 2009 WHO dengue classification scheme: dengue without warning sign symptoms (A), dengue with warning signs (B) and severe dengue (C). Colored shadings represent the different phases of dengue fever. Horizontal dotted line represents healthy mean average. Mean ± SEM.

**Figure 4:**
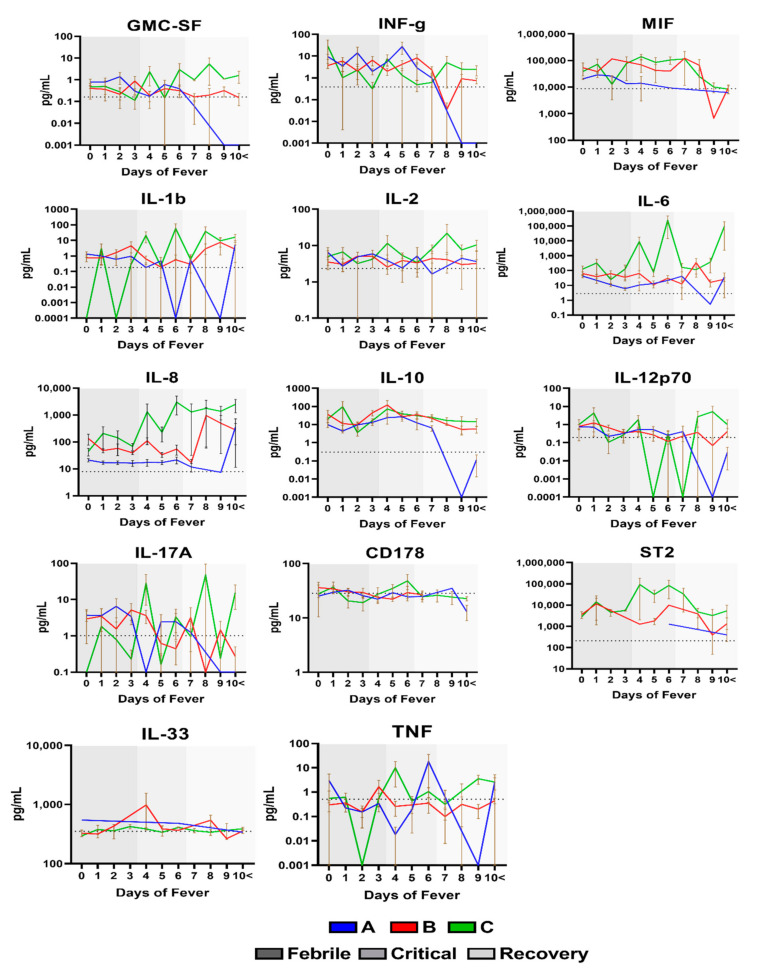
The level of Pro and Anti-Inflammatory molecules in dengue patients at different days of fever Graphs representing the levels between the different groups of DENV patients previously divided following the 2009 WHO dengue classification scheme: dengue without warning sign symptoms (A), dengue with warning signs (B) and severe dengue (C). Colored shadings represent the different phases of dengue fever. Horizontal dotted line represents healthy mean average. Mean ± SEM.

To better observe the overall changes of individual cytokines, the expression profile of individual cytokines for different days of fever and disease severity were plotted—Supplementary Figure S3. It was observed that all the cytokines concentration fluctuated significantly in the different groups and over the days of fever as reflected by the mean and graphs—Table 1. Vascular damage and hemorrhage are the hallmark features of increased vascular permeability in dengue patients. Clinical evidence suggests that vascular damage plays a key role in the pathophysiology of dengue hemorrhagic fever (DHF). During disease progression, infected cells produce inflammatory cytokines such as TNF-α, IL-6, and IL-10. As a result of this stimulation, endothelial cells increase the expression of adhesion molecules such as CD62E (E-Selectin), CD106 (VCAM-1) and CD62P (P-Selectin) which leads to local inflammation, endothelial damage and plasma leakage [25,26,27]. Hence, our results demonstrated that adhesion molecules like CD54, CD106, CD62E, CD62L, CD62P and CD154, based upon the days of fever, all exhibited this fluctuation in their expression starting from day 0 when compared to the healthy control mean (baseline) but become more normal (baseline) in the recovery phase after day 8 Figure 3. Of note, the levels of pro-inflammatory cytokines like GM-CSF, IL-2, IL-6, IL-8 remained at an all-time high especially during the critical phase of the disease, CD178 remained at an all-time low Figure 4. On the other hand, cytokines like MIF, IL-10, and IL-33 peaked during the critical phase of the disease but became more normalized at the recovery phase after day 8 Figure 4. IL-1b, IL-12p70, IL-17A, TNF and ST2 showed no visible trend and fluctuated between the days of fever Figure 4.

### 2.5. Potential Biomarker for Dengue Fever Diagnosis and Predictive Marker of Dengue Severity

**Figure 5:**
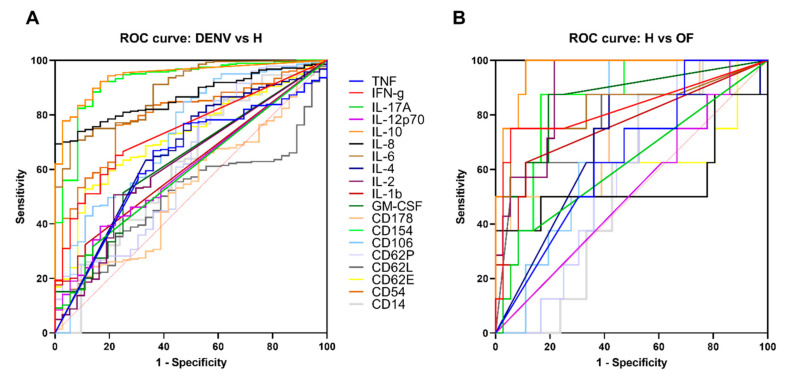
ROC curve of cytokines. Univariate logistic regression analysis was conducted. Performance of ROC curves of TNF, IFN-g, IL-17A, IL-12p70, IL-10, IL-8, IL-6, IL-2, IL-2, IL-1b, GM-CSF, CD178, CD154, CD106, CD62P, CD62L, CD62E, CD54 and CD14 for predicting DENV and differentiating from Healthy (H) and other fever (OF). Plots depict he tradeoff between sensitivity and specificity. The closer the curve follows the left-hand border and the top border of the ROC space, the more accurate the test. (**A**) ROC curve compared between DENV patients and healthy subjects. (**B**) ROC curve compared between healthy subject and other fever patients.

To possibly determine a potential cytokine to serve as a diagnostic tool for dengue virus, ROC curves were drawn for DENV, H and OF groups. The ROC curve was plotted only for cytokines that showed a significant difference in statistical analysis between the three groups. IL-33 was excluded from the ROC curve due to the OF group not having data, also CD14, CD12b and ST2 for not showing any significant differences in statistical analysis between the groups. Interestingly, the area under ROC curve of IL-10 proved to be the largest among all cytokines. A concentration of >0.499434 pg/mL was the optimal cutoff value for distinguishing between the H and DENV group (AUC = 0.944, Sensitivity = 89.79, Specificity = 86.11), while a concentration of >0.950302 pg/mL was the optimal cutoff value (AUC = 0.969, Sensitivity = 87.5, Specificity = 88.89) for distinguishing between the H and OF group Figure 5A,B and Supplementary Table S1.

**Figure 6:**
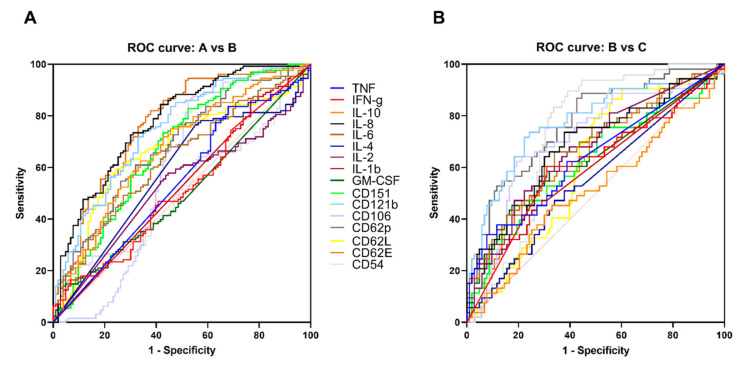
ROC curve of cytokines. Univariate logistic regression analysis was conducted. Performance of ROC curves of TNF, IFN-g, IL-17A, IL-12p70, IL-10, IL-8, IL-6, IL-2, IL-2, IL-1b, GM-CSF, CD178, CD151, CD121b, CD106, CD62P, CD62L, CD62E and CD54 for predicting DENV severity and differentiating from dengue without warning sign symp-toms A, dengue with warning signs B and severe dengue C. Plots depict he tradeoff between sensitivity and speci-ficity. The closer the curve follows the left-hand border and the top border of the ROC space, the more accurate the test. (**A**) ROC curve compared between dengue without warning sign symptoms and dengue with warning signs. (**B**) ROC curve compared between dengue with warning signs and severe dengue.

To further evaluate the diagnostic potential marker for distinguishing between the different disease severity, ROC curves were drawn. The ROC curve was plotted only for cytokines that showed significant differences in statistical analysis between the three groups. CD14, CD178, CD12p70, IL-17A, IL-33, MIF and ST2 were excluded from the ROC curve for not showing any significant differences in statistical analysis between the groups. It was observed that the area under ROC curve for CD121b did a better job in discriminating between the disease severity Figure 6A and Supplementary Table S1. A concentration of ≤1564.64 pg/mL was the optimal cutoff value (AUC = 0.744, Sensitivity = 71.87, Specificity = 66.99) for distinguishing between the A and B group, while a concentration of >4418.043 pg/mL was the optimal cutoff value (AUC = 0.775, Sensitivity = 71.70, Specificity = 77.67) for distinguishing between the B and C group Figure 6A,B, and Supplementary Table S1.

## 3. Discussions

In this study, we compared twenty-three different cytokines profile pattern, namely IL-1b, IL-2, IL-4, IL-6, IL-8, IL-10, IL-12p70, IL-17A, IL-33, CD14, CD54, CD62E, CD62L, CD62p, CD106, CD121b, CD154, CD178, GM-CSF, IFN-g, MIF, ST2 and TNF of dengue patients at different severity of the disease, with the aim to understand their profile during different phases of dengue and their possible association with the disease severity.

Dengue exhibits a wide spectrum of clinical features that usually start to be prominent after an incubation period of approximately 3–10 days, but the majority of cases are asymptomatic [28]. Apart from the wide spectrum of symptoms, clinician and health care services face a huge challenge during an outbreak that sometimes lead to poor prognostics and diagnosis in being able to differentiate between individuals who have dengue, other fevers or even chikungunya. For example, during an outbreak, misdiagnosis is common especially in countries that lack screening devices. Clinicians tend not to confirm their diagnosis in the laboratory since when working off symptoms alone, common flu, other fevers and dengue fever are extremely similar in the acute stages, therefore DENV infection is not assumed masking the spread of the disease [8,29]. Our study adds more to our already known knowledge and widens our diagnostic toolbox of cytokines for potentially describing a novel panel of clinically informative biomarkers to distinguish dengue fever from other fever. There was a significant difference between DENV, H and OF groups for cytokines CD154, GM-CSF and IL-2, suggesting that they can be used as markers for distinguishing between dengue fever and OF Figure 1. To further explore a potential cytokine to serve as a diagnostic tool for the dengue virus, ROC curves were drawn for DENV, H and OF groups. Interestingly, the area under ROC curve of IL-10 proved to be the largest among all cytokines. A concentration of >0.499434 mg/mL was the optimal cutoff value for predicting dengue fever (Sensitivity = 89.79, Specificity = 86.11) Figure 5A,B and Supplementary Table S1. IL-10 is a cytokine with pleiotropic effects in immunoregulation and inflammation, including the inhibition of immune mediator secretion, antigen presentation and phagocytosis [30]. Over the years, evidence has shown that microbes such as fungi, bacteria and viruses can regulate the host cell IL-10 expression that would allow persistent infection. There has also been an emerging role of IL-10 in dengue virus infection associating it with enhancing the infection severity and contributing to the pathogenesis of dengue infections by inhibiting DENV-specific T cell responses or downstream signaling; however the mechanism for such responses are in need of further investigation [30,31].

Compared with dengue without symptoms and dengue with symptoms, patients suffering from severe dengue often have poorer outcomes and a higher mortality rate. Recently, severe dengue clinical data of signs and symptoms have shown that bleeding, vomiting, nausea, skin rash, hepatosplenomegaly and abdominal pain are associated with severe dengue causing an estimated 500,000 people requiring hospitalization each year and having an estimated 2.5% case fatality, annually [4,32]. Therefore, identifying an ideal biomarker would not only allow us to identify the individuals who would be at great risk of developing severe dengue but more importantly allow clinicians to make early intervention and quicker diagnosis of the disease severity. Numerous studies have demonstrated evidence to support the cytokine storm theory which is a role for cytokines where the concentrations of cytokines, mediators and soluble receptors may be significantly disrupted during dengue infection [33,34]. Even though progress has been made in recent years in trying to understand the mechanisms of cytokine production during the disease severity, our understanding remains incomplete, especially due to conflicting results in the cytokine levels observed by different studies [20]. Although the conflicting results are likely due to differences in the study design, the way the samples are processed, the timing of sample collection, and importantly due to the differences in the study cohort, the possibility that different pathways could lead to similar clinical manifestations is also considered [20].

In this study, we also provide evidence that cytokine dysfunction reflected by cytokine storm could contribute to the disease severity. Our observations demonstrated that some cytokines fluctuated dramatically while some exhibited a pattern in the early illness phase and maintained a pattern as the illness progressed. Cytokine expression profile of dengue patients at different severity of the disease showed that almost all cytokines expression levels were increased when compared to the healthy control mean (baseline) Figure 2. Previous research has demonstrated that cytokines such as TNF, IFN-g, GM-CSF, IL-1b, IL-6, IL-8, IL-10 and ST2 are found to be related to plasma leakage in DHF/DSS and can serve as a biomarker but have failed to explain disease severity in many of the cases [6,35,36,37,38,39]. Most of our results were consistent with other findings, showing a significant difference compared to the healthy control. Increased levels of IFN-g, TNF, IL-1b, IL-6, IL-8, IL-10 and GM-CSF were also observed in our data coinciding with other findings that have been observed to increase in the DHF when compared to DF [40,41]. Interestingly, the area under ROC curve of CD121b did a better job in discriminating between the disease severity Figure 6A,B and Supplementary Table S1. Of note, it was also observed that CD121b was not only significantly different by statistical analysis among the different groups but also showed an increased pattern expression along with the disease severity and days of fever Figure 2 and Supplementary Figure S3. To our knowledge, this is the first time CD121b is observed as a potential biomarker for distinguishing between the disease severity. A concentration of >4418.043 pg/mL was the optimal cutoff value (Sensitivity = 71.70, Specificity = 77.67) for distinguishing between the A and B group, while a concentration of >0.499434 pg/mL was the optimal cutoff value (Sensitivity = 89.79, Specificity = 86.11) for distinguishing between the B and C group Figure 6B, and Supplementary Table S1.

CD121b, also known as IL-1R2 is an interleukin receptor that acts as a decoy receptor and as a negative regulator for certain cytokines that belong to the interleukin-1 receptor family (IL-1). This protein binds interleukin-1α (IL1A), interleukin-1β (IL1B), and interleukin 1 receptor antagonist (IL1RA), preventing them from binding to their regular receptors (interleukin receptor type 1, IL-1R1) and thereby inhibiting the transduction of their signaling or hinder the signaling complex assembly. The IL-1 family of cytokines and receptors is unique in immunology because the IL-1 family and Toll-like receptor (TLR) families share similar functions, they play a central role in the regulation of immune and inflammatory response [42,43]. Over the years, surfacing research show that the IL-1 family plays a major role as a proinflammatory cytokine in cancer, heart conditions and various immune diseases, but its role with CD121b is not fully understood [44,45]. CD121b is expressed by neutrophils, T cells and mainly expressed by myeloid cells. Non-immune cells like keratinocytes and some types of epithelial cells have also been observed to express CD121b under normal conditions, but increases upon inflammation [46,47]. Moreover, recent publications have further demonstrated the crucial role of CD121b and inflammation in Alzheimer’s patients, sepsis, Hodgkin lymphoma and IgG4-related disease [48].

There are certain limitations in our study, one of the limitations was that we could not identify the previous infection history of the patients to see whether this was a secondary infection. Taiwan had experienced two other dengue outbreaks before the 2015 Taiwan (Tainan) outbreak: the first in Penghu County in 2011 and the second being in Kaohsiung City in 2014 [49]. Hence it is logical to speculate that probably some of the cases were secondary infection and could lead to a difference in results. Another limitation in our study involves the low number of volunteers in the Healthy control group and OF group.

## 4. Materials and Methods

### 4.1. Ethical Statement and Study Cohort

In this study, a total of 328 cytokine levels were observed and analyzed from 243 patients’ sera. The total 328 data points analyzed consisted of measurements taken from patients who had single or multiple admission to the hospital, as well as measurements taken at different days of fever. Samples with written informed consent were obtained from patients as they were admitted to National Cheng Kung University Hospital between July and October during the 2015 Taiwan dengue outbreak (IRB #B-ER-104-178). To maintain the confidentiality of samples, clinical data were recorded with a serial ID in the report. Patients that were found to be positive for DENV-2 were classified into 3 groups according to the day of illness and clinical symptoms following the 2009 WHO dengue classification scheme [21,50]. These categories included, dengue without warning sign symptoms (A) which consisted of 128 patients; dengue with warning signs which consisted of 103 (B) and severe dengue (C) which consisted of 53 patients. Eight patients who had fever but were dengue negative were classified as other fever (OF). The healthy (Healthy) control group consisted of 36 patients. Data characteristics of the patient such as age, cytokine level and general statistics in this study were provided in Table 1. This is a retrospective study and complete demographic descriptions of the enrolled patients had been previously reported [49].

### 4.2. Dengue Confirmatory Tests

These samples collected during 2015 dengue outbreaks in Taiwan have been reported previously [49]. Some of the specimens were reaffirmed by SD BIOLINE Dengue Duo Rapid Test kit (Abbott, Chicago, IL, USA) and by plaque assay for the viral titers in these specimens. Patients that were dengue negative were categorized as other fever (OF) in this experiment. Furthermore, those who presented symptoms such as cough, obstructions, other un-wellness, or immunocompromised disease were excluded from the healthy control group.

### 4.3. Cytokine Identification and Quantification

Cytometric Bean Assay (CBA) (BD Biosciences, Franklin Lakes, NJ, USA) was used to quantify the cytokine levels in each sample. Twenty-three different cytokines, namely IL-1b, IL-2, IL-4, IL-6, IL-8, IL-10, IL-12p70, IL-17A, IL-33, CD14, CD54, CD62E, CD62L, CD62p, CD106, CD121b, CD154, CD178, GM-CSF, IFN-g, MIF, ST2 and TNF were evaluated simultaneously in each sample. The patient’s plasma was used to analyze these cytokines levels using BD LSRFortessa (BD Biosciences, Franklin Lakes, NJ, USA). In summary, 30 μL of the patient’s plasma was diluted with sample buffer from the cytometric bead array (CBA) kit following to the manufacturer’s instruction, followed by mixing it with specific beads conjugated with specific antibodies (Abs) which would exhibit a specific fluorescent intensity in proportion to the number of bound analytes when being analyzed using the BD LSR Fortessa.

### 4.4. Statistical Analysis

All raw data were stored in a computerized database (MS Excel 2016, Microsoft, Redmond, Washington, USA). Descriptive statistics were calculated for all patients in this study and summarized in Table 1. Statistical Analysis was performed using R- Studio v1.2.5042 (RStudio: Integrated Development for R. RStudio, Boston, MA, USA) and Graph-Pad Prim v7 (Graphpad Software, San Diego, CA, USA) Kruskal–Wallis test was performed on all groups, whereas the Mann–Whitney test was used to compare groups head to head. Boxplots and scatter plots were used to visualize the distribution of the cytokine data within the different groups or for the different days of fever. A *p*-Value < 0.05 was considered as significantly different by statistical analysis. Receiver operating characteristic (ROC) curves were used to assess the diagnostic values in discriminating dengue fever and disease severity. The optimal diagnostic cutoff value was determined according to Youden’s J-statistic, and the relative sensitivity, specificity, positive and negative predictive values were calculated.

## 5. Conclusions

In conclusion, these observations from previous research and our work further provide new insight and evidence on the possible role of CD121b in DENV infection and its potential to serve as a diagnostic marker and warrants further investigation. In our study, we found that the IL-10 could be a potential diagnostic marker for DF and CD121b a potential predictor marker for the disease severity based upon the cut-off value in ROC curve. However, one parameter alone cannot be exclusively utilized as a marker to predict DF or the disease severity. Hence, the current findings can provide key information in understanding and exploring possible predictors of severe dengue and its progression in patients without any warning signs who might later develop to severe dengue. In addition, the suggested panel of cytokines in the study, along with already investigated sources add more to our understanding of the role of cytokines during DENV. It is possible to implement these cytokines in clinical practice to improve patients’ supportive care.

## Figures and Tables

**Table 1:** Study Cohort Summary and laboratory parameters. A, dengue without warning symptoms; B, Dengue with warning signs; C, Sever Dengue; OF, other febrile infection; Healthy, Healthy; CD14, cluster of differentiation; CD54, cluster of differentiation 54; CD62E, E-selectin;CD62L, L-selectin; CD62p, P-selectin; CD106, cluster of differentiation 106; CD121B, Cluster of Differentiation 121 β; CD154, Cluster of Differentiation 154; CD178, cluster of differentiation 178; GM-CSF, granulocyte-macrophage colony-stimulating factor; IL-1b, Interleukin 1 beta; IL-2, interleukin 2; L-4, interleukin 4; IL-6, interleukin 6; IL-8, interleukin 8; IL-10, interleukin 10;IL-12p70, interleukin 12p70; IL-17A, interleukin 17A; IL-33, interleukin 133; IFN-g, interferon gamma; MIF, macrophage migration inhibitory factor; ST2,TNF, tumor necrosis factor. Cytokine concentration in pg/mL. Kruskal–Wallis test and Mann–Whitney test for continuous variable not-normally distributed; mean ± SD; number (percentage of total); median (minimum–maximum); N.D (no data).

Demographics	A	B	C	Healthy	OF	*p* Value	SD
*Total*	128(39%)	103(31%)	53(16%)	36(11%)	8(3%)		
*Age (years)*	(54)18–86	(67)26–93	(71)55–84	(47)22–80	(51)24–83		
*IL-1b*	0.9585 ± 2.941	1.623 ± 5.493	17.65 ± 66.62	0.1789 ± 0.5579	1.13 ± 1.387	<0.0165	b,c,d,e,f,h,j
*IL-2*	4.593 ± 9.347	3.693 ± 4.587	7.91 ± 10.97	2.327 ± 4.03	12.55 ± 9.966	<0.0174	b,d,e,f,g,h,j
*IL-4*	24.79 ± 46.56	46.32 ± 108.4	59,499 ± 293,443	2.827 ± 3.435	214.8 ± 563.7	<0.0357	a,b,c,e,f,g,h,i
*IL-8*	0.2349 ± 0.7667	0.2769 ± 1.516	0.208 ± 0.3771	7.89 ± 3.067	38.69 ± 67.47	<0.0132	a,b,c,e,f,h,i,j
*IL-6*	26.24 ± 93.67	135.9 ± 417.3	1447 ± 3351	0.3075 ± 0.8265	16.86 ± 25.73	<0.0086	a,b,c,f,h,j
*IL-10*	10.34 ± 17.78	34.05 ± 116.4	32.01 ± 65.55	0.2933 ± 0.324	0.1373 ± 0.3502	<0.0003	c,f
*IL-17A*	0.5347 ± 1.192	0.513 ± 0.7812	1.227 ± 3.232	0.1923 ± 0.3936	0.08852 ± 0.1501	<0.0381	c,f,h
*IL-12p70*	3.568 ± 10.31	2.271 ± 5.649	10.12 ± 30.76	1.009 ± 3.006	3.164 ± 4.554	<0.0335	a,b,c,f,h
*IL-33*	454.1 ± 103.9	452.3 ± 384.9	378 ± 73.81	350.4 ± 86.95	N.D	NS	
*CD14*	35,861 ± 35,861	74,193 ± 207,439	82,228,957 ± 402,093,646	305,911 ± 1,174,615	37,812 ± 16,759	<0.0429	c,f
*CD54*	6,033,111 ± 30,288,666	698,840,332 + 6,609,196,581	136224 ± 141633	108,486 ± 143,675	1,305,628 + 2,110,778	<0.0182	b,c,e,f,h,i,j
*CD62E*	4644 ± 3360	258,653 ± 2,538,327	16535 ± 28714	3916 ± 2426	6160 ± 4018	<0.0104	a,b,e,f,h,i
*CD62L*	1,415,889 ± 13,713,537	524,272 ± 3,791,122	5,005,464 ± 35,347,942	166,876 ± 680,047	162,093 ± 430,665	<0.0114	a,b,c,e,g,h,i,j
*CD62P*	6833 ± 4593	10,865 ± 11,116	17,773 ± 10,290	7198 ± 4672	7610 ± 3141	<0.0025	a,b,e,f,h,i
*CD106*	7916 ± 14,402	18,292 ± 34,423	38,789 ± 36,740	54,096 ± 50,677	6913 ± 2126	<0.0333	a,b,c,e,f,i,j
*CD121b*	1539 ± 1277	3229 ± 2771	7111 ± 4478	2970 ± 2284	1286 ± 690.5	<0.003	b,c,e,f,i,
*CD154*	537.3 ± 424.6	357.4 ± 371.8	278.2 ± 5375.9	1682 ± 1039	703.2 ± 432.8	<0.0088	a,b,c,e,f,g,h,i,j
*CD178*	26.69 ± 10.29	28.46 ± 17.89	29.1 ± 21.27	28.32 ± 12.67	17.4 ± 9.591	<0.0358	d,g,i.j
*GM-CSF*	0.7341 ± 2.091	0.3437 ± 0.7958	1.57 ± 3.986	0.1625 ± 0.3137	1.574 ± 2.119	<0.0156	b,c,d,e,f,g,h,j
*IFN-g*	8.178 ± 26.47	3.526 ± 6.077	3.337 ± 8.582	0.3811 ± 1.054	3.965 ± 4.153	<0.0117	b,c,e,f,h,j
*MIF*	21,536 ± 11,198	46,859 ± 47,061	60,101 ± 71,487	8769 ± 2618	N.D	<0.0387	c,f,h
*ST2*	838.2 ± 623.9	3906 ± 5272	26998 ± 65830	210.5 ± 264.9	N.D	<0.0047	f,h
*TNF*	2.061 ± 13.91	0.4251 ± 1.441	2.232 ± 7.317	0.5135 ± 0.5248	0.2321 ± 0.2905	<0.0031	b,c,e,f

Significant difference (S.D): (a) between A and B; (b) between A and C; (c) between A and Healthy; (d) between A and OF; (e) between B and C; (f) between B and Healthy; (g) between B and OF; (h) between C and Healthy; (i) between C and OF; (j) between Healthy and OF.

## Data Availability

The study did not report any data.

## Supplementary Materials

The following are available online at https://www.mdpi.com/1422-0067/22/6/2879/s1, Figure S1: The levels of cytokines in DENV patients, Figure S2: The levels of cytokines in DENV patients with different severity, Figure S3: The levels of cytokines in DENV patients at different days of fever, Table S1: Area under the curve of cytokines describing the performances of cytokines in discriminating: DENV- differentiating from Healthy (Healthy) and other fever (OF); DENV severity- differentiating from dengue without warning sign symptoms (A), dengue with warning signs (B) and severe dengue (C).
