# Supplementary material for: Cytokine Signature of Dengue Patients at Different Severity of the Disease
Source: Int J Mol Sci. 2021 Mar 12;22(6):2879. doi: 10.3390/ijms22062879 (PMC7999441; doi:10.3390/ijms22062879)
Supplement: Supplementary file 1 [file ijms-22-02879-s001.pdf]

Supplementary Data

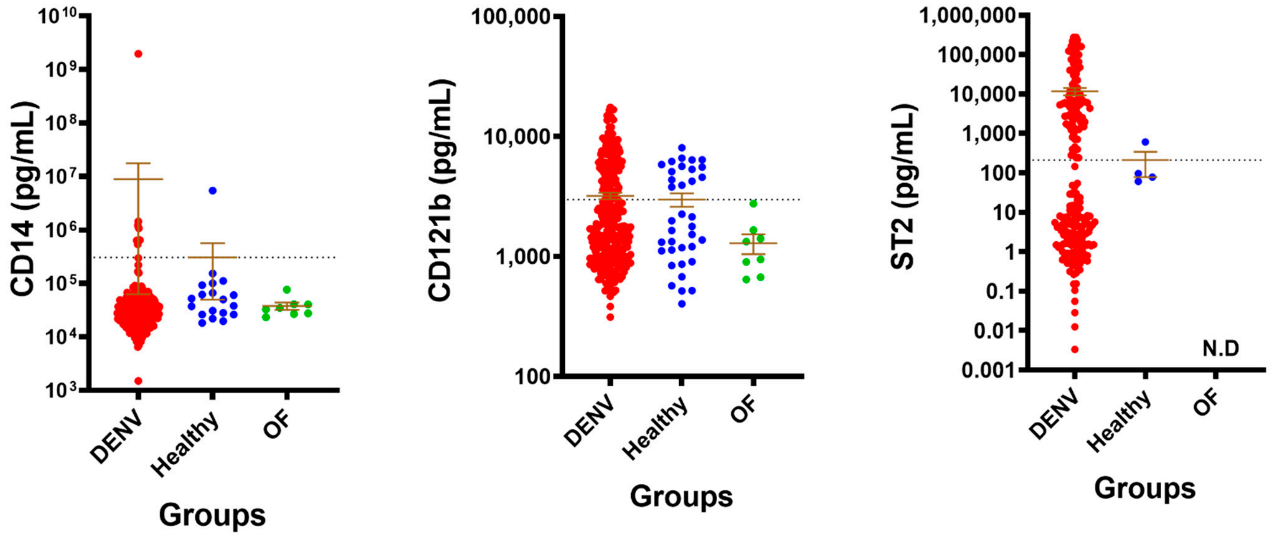

Supplementary Figure S1. The levels of cytokines in DENV patients. Patients were divided into three groups: Laboratory confirmed DENV-2 patients (DENV), laboratory confirmed DENV negative (OF) and healthy volunteers (Healthy). Only cytokines having a significance difference between either DENV, Healthy or OF were shown in Figure 1. Mean ± SEM. Horizontal dotted line represents health mean average. N.D (no data). *p*<0.05, *; <0.01, **; <0.001, ***.

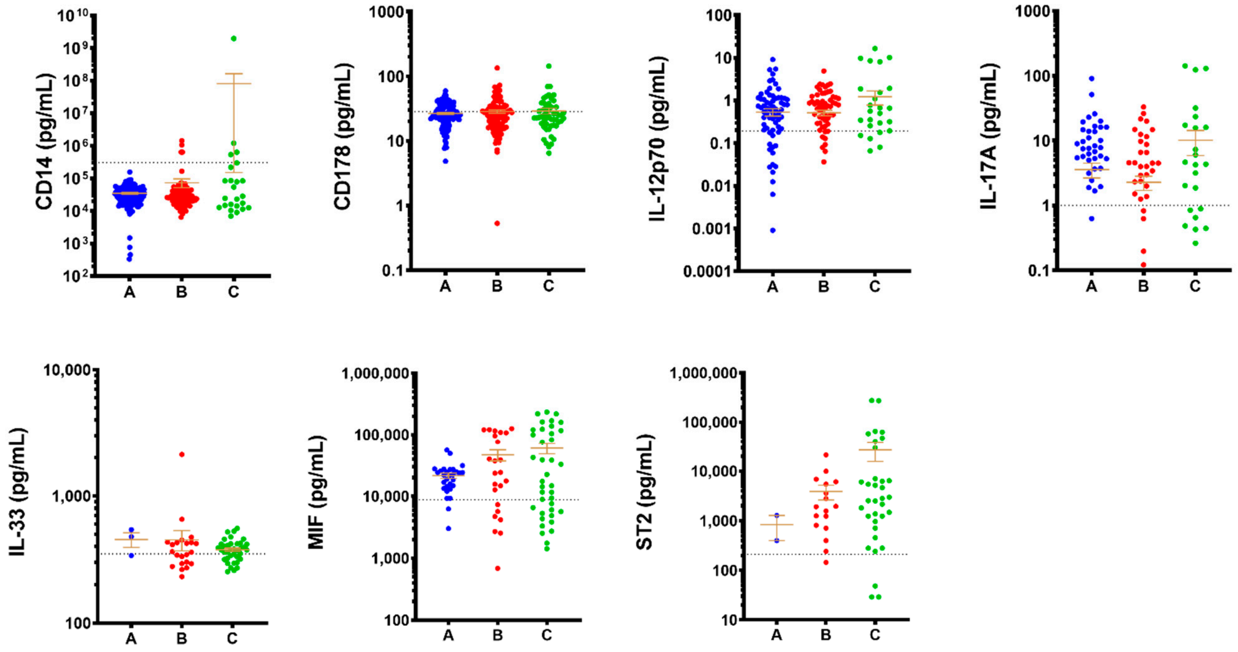

Supplementary Figure S2. The levels of cytokines in DENV patients with different severity. DENV patients were divided into three groups according to the day of illness and clinical symptoms following the 2009 WHO dengue classification scheme: dengue without warning sign symptoms (A), dengue with warning signs (B) and severe dengue (C). Only cytokines having a significance difference between either A, B or C in Figure 2. Mean ± SEM. Horizontal dotted line represents health mean average. p<0.05, *; <0.01, **; <0.001, ***.

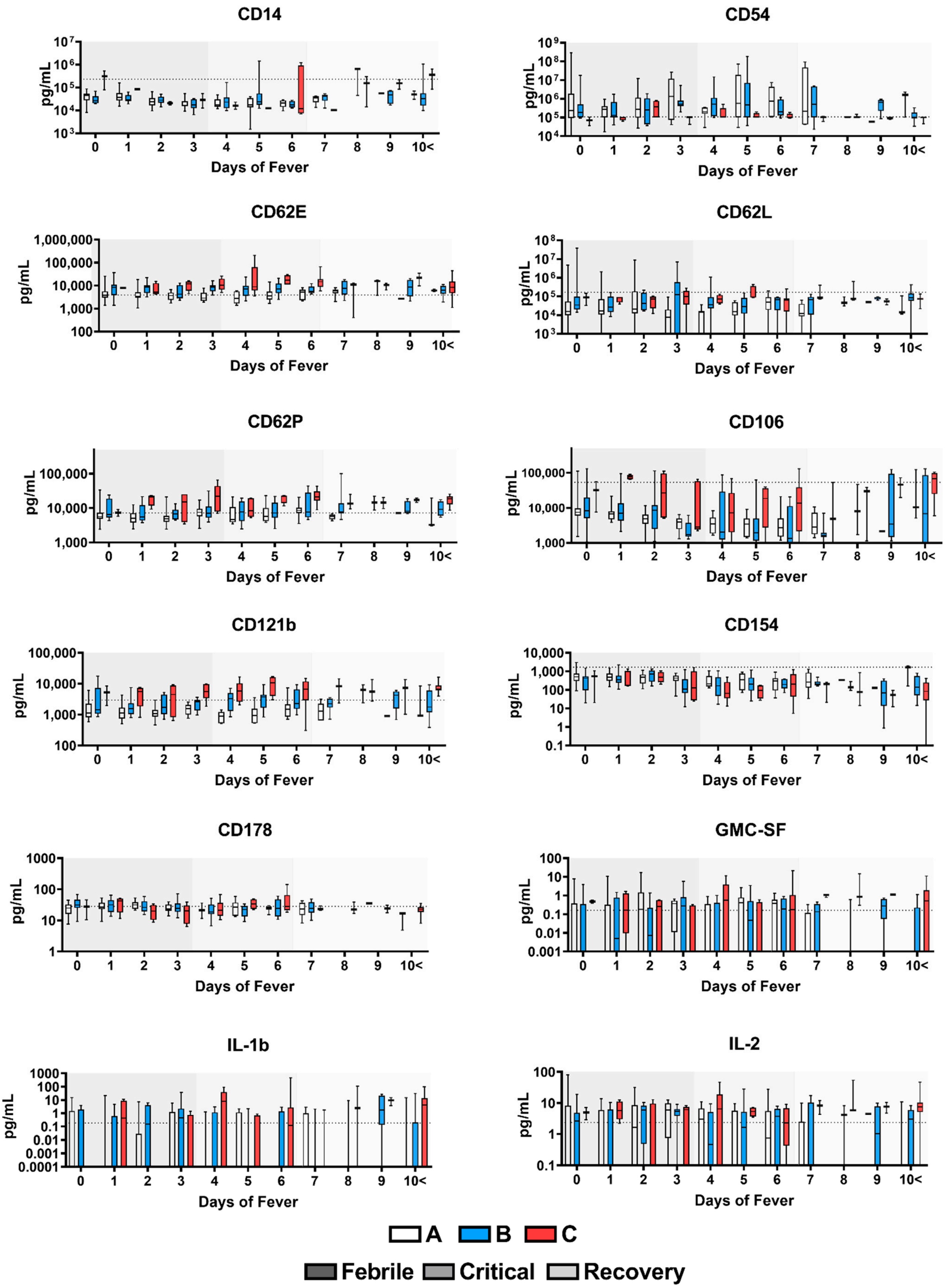
CD14
CD54
CD62E
CD62L
CD62P
CD106
CD121b
CD154
CD178
GMC-SF
IL-1b
IL-2
pg/mL
Days of Fever
0
1
2
3
4
5
6
7
8
9
10<
A
B
C
Febrile
Critical
Recovery

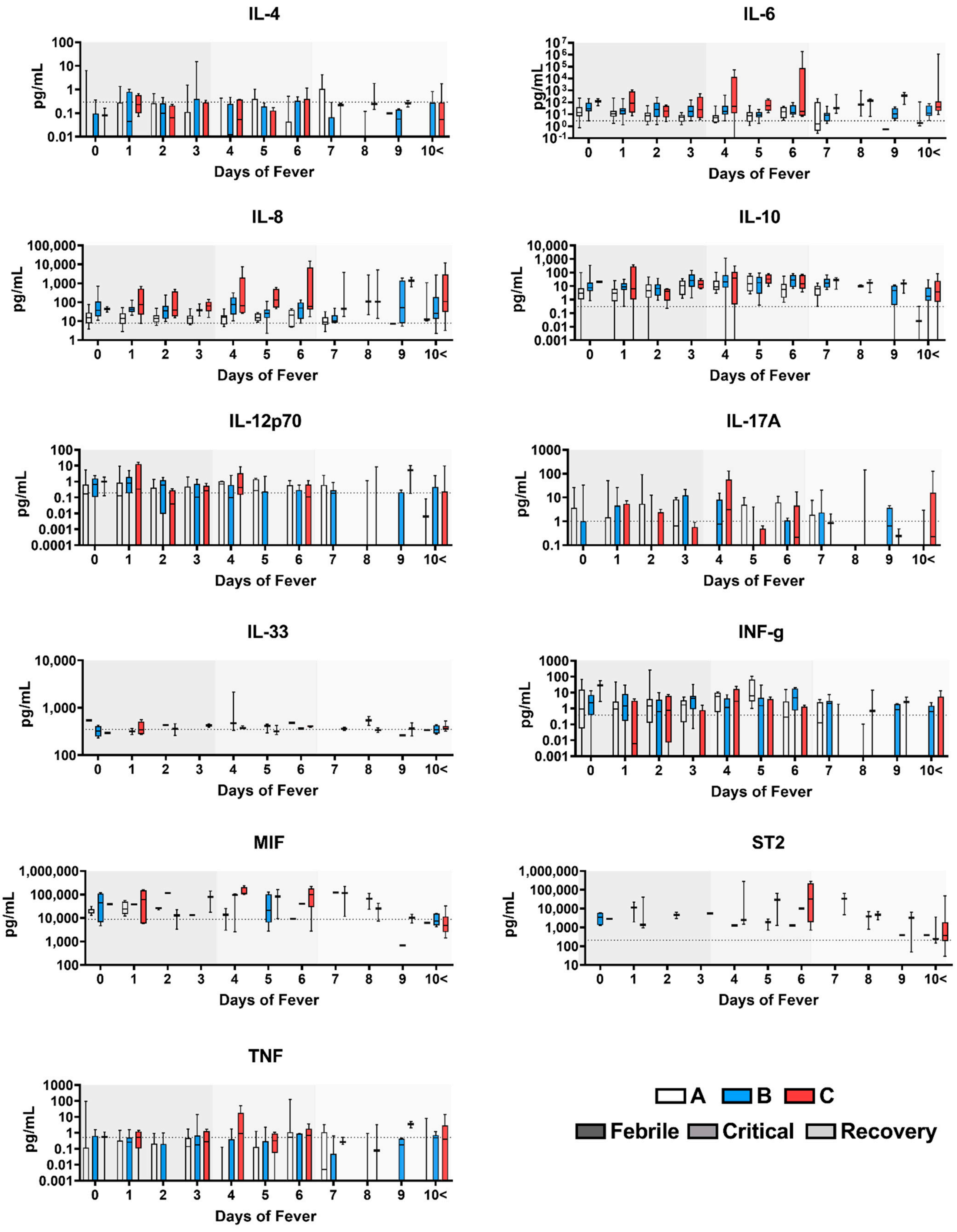
IL-4
IL-6
IL-8
IL-10
IL-12p70
IL-17A
IL-33
INF-g
MIF
ST2
TNF
pg/mL
Days of Fever
0 1 2 3 4 5 6 7 8 9 10<
A
B
C
Febrile
Critical
Recovery

Supplementary Figure S3. The levels of cytokines in DENV patients at different days of fever. DENV patients were divided into three groups according to the day of illness and clinical symptoms following the 2009 WHO dengue classification scheme: dengue without warning sign symptoms (A), dengue with warning signs (B) and severe dengue (C). The patient's cytokine level was monitored during the febrile, critical and recovery phase of dengue. Each cytokine was plotted according to the day of fever and disease severity.

	AUC	Criterion	Sensitivity	95% CI	Specificity	95% CI	+LR	-LR
	**IL-10**							
Healthy vs DENV	0.944	>0.499434	89.79	85.7 - 93.1	86.11	70.5 - 95.3	6.46	0.12
Healthy vs OF	0.969	>0.950302	87.5	47.3 - 99.7	88.89	73.9 - 96.9	7.87	0.14
	**CD121b**							
A vs B	0.744	≤1564.64	71.87	63.2 - 79.5	66.99	57.0 - 75.9	2.18	0.42
B vs C	0.775	>4418.043	71.7	57.7 - 83.2	77.67	68.4 - 85.3	3.21	0.36

Supplementary Table S1. Area under the curve of cytokines describing the performances of cytokines in discriminating: DENV- differentiating from Healthy (Healthy) and other fever (OF); DENV severity-differentiating from dengue without warning sign symptoms (A), dengue with warning signs (B) and severe dengue (C).
